# Supplementary material for: Chitosan Functionalized with 2-Methylpyridine Cross-Linker Cellulose to Adsorb Pb(II) from Water
Source: Polymers (Basel). 2021 Sep 18;13(18):3166. doi: 10.3390/polym13183166 (PMC8469900; doi:10.3390/polym13183166)
Supplement: Supplementary file 1 [file polymers-13-03166-s001.zip › polymers-1366601-supplementary.pdf]

Supplementary Materials

Figure S1. Spectrum FTIR before and after lead adsorption of Com-1 and Pb-Com1

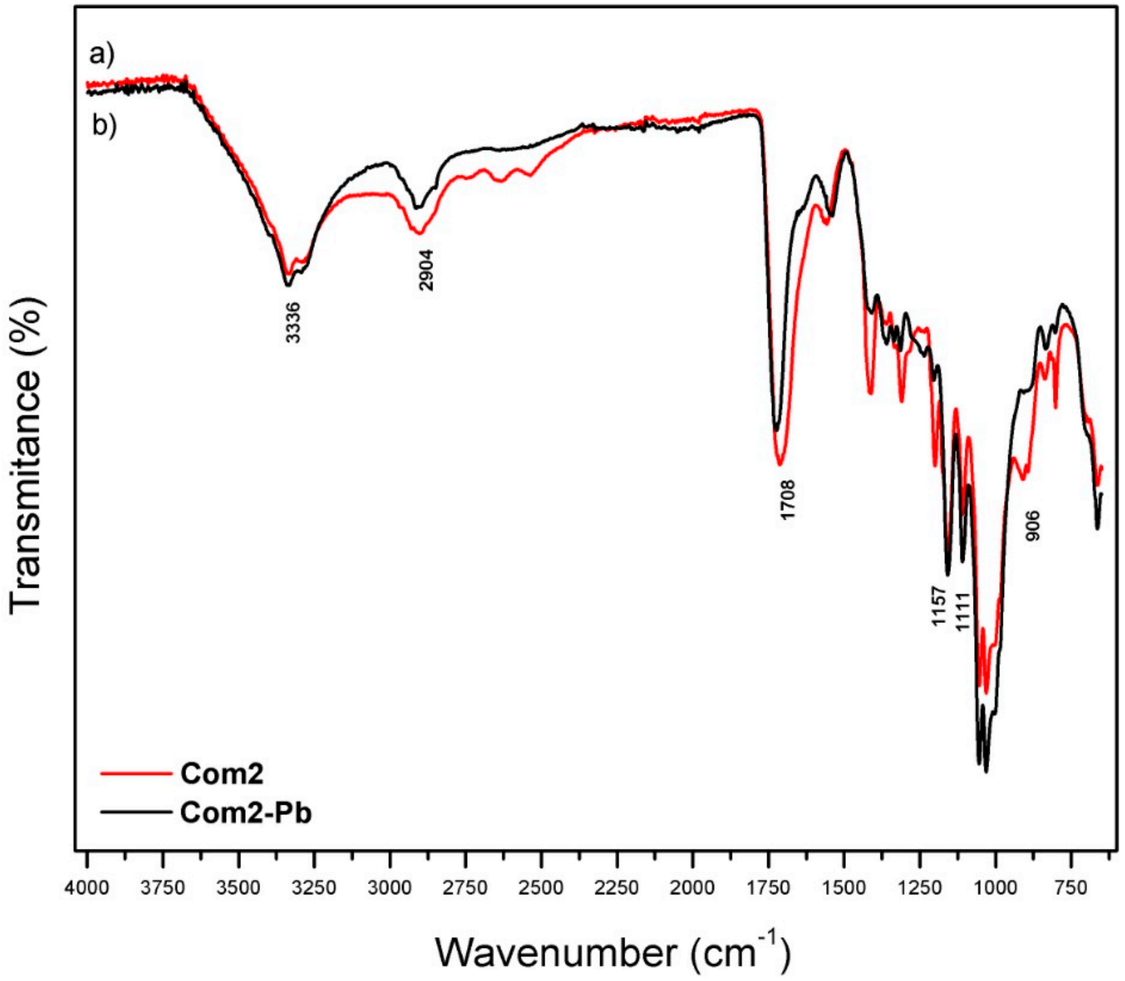

Figure S2. Spectrum FTIR before and after lead adsorption of Com-3 and Pb-Com-3

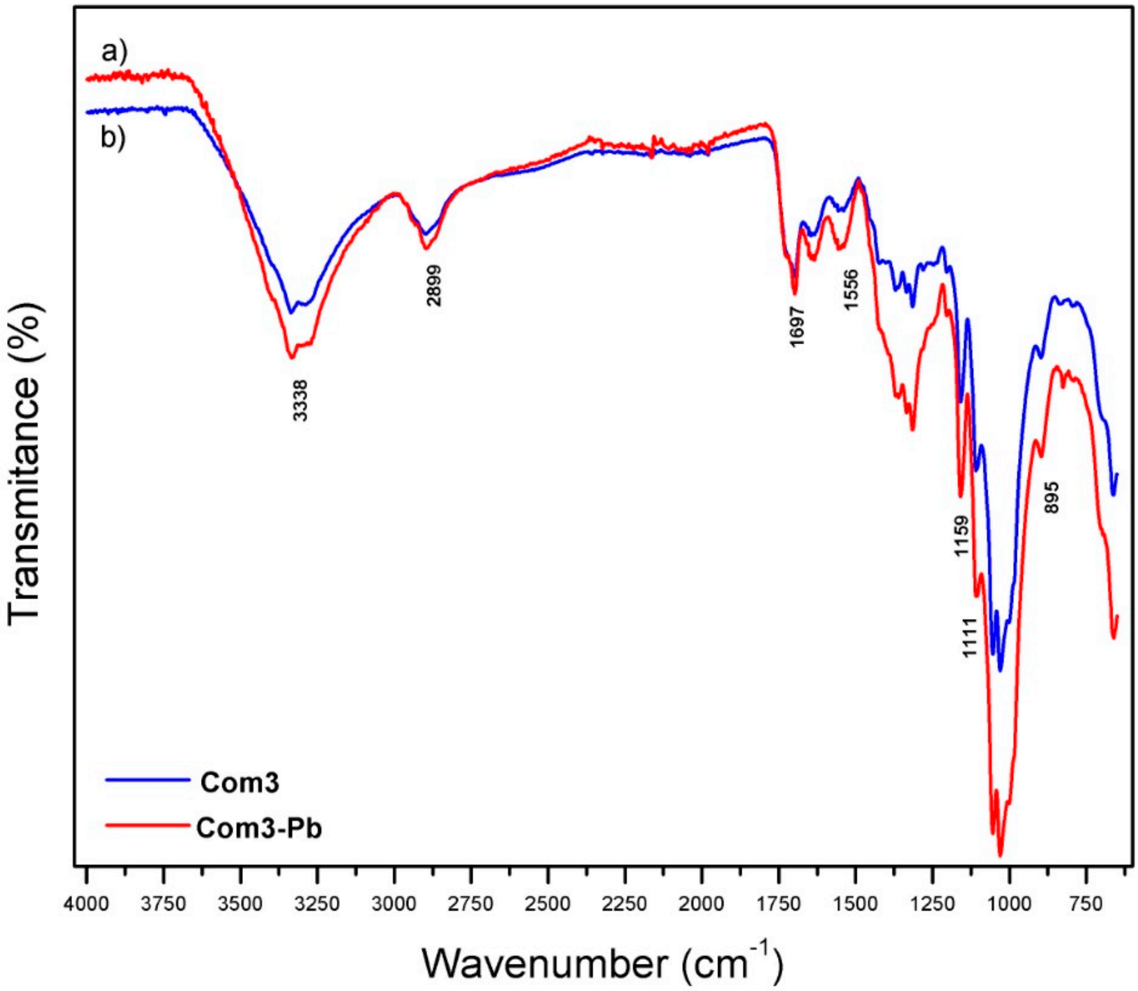

Table S1. Relative atomic concentration (%) for the different composites **before** lead adsorption.

	C 1*s*				N 1*s*				Pb 4*f*		
Material	C-C/ C-H	C-OH/C-O	O-C-O	O=C-O/ C=O	N-py	$-NH_2$	O=C-NH-	$-NH_3^+$	Pb-O	$Pb-NO_3$	O-[illegible]
Cellulose	40.98	25.83	5.79	1.33	0.00	0.16	0.44	0.00	0.00	0.00	4.9[illegible]
Com1	37.31	19.75	3.17	10.62	0.00	0.28	0.37	0.02	0.00	0.00	5.1[illegible]
Com3	16.89	32.62	10.47	2.74	0.00	1.16	2.70	1.41	0.00	0.00	5.8[illegible]
Com4	19.17	32.30	10.10	4.38	0.02	0.16	0.45	0.00	0.00	0.00	7.4[illegible]

Table S1 show the atomic concentration of different functional groups. The main differences between then is related to the attenuation signal attributed to lea
